# Distribution and clinical significance of hepatitis B virus genotypes in Pakistan

**DOI:** 10.1186/s12876-016-0513-5

**Published:** 2016-08-26

**Authors:** Majid Mahmood, Muhammad Asim Anwar, Azra Khanum, Nasib Zaman, Abida Raza

**Affiliations:** 1 Department of Zoology, The University of Poonch, Rawalakot, Azad Jammu and Kashmir 12350 Pakistan; 2 Department of Zoology, Pir Mehr Ali Shah Arid Agriculture University, Rawalpindi, 46300 Pakistan; 3 Department of General Medicine, Pakistan Atomic Energy Commission (PAEC) General Hospital, Islamabad, 44000 Pakistan; 4 Barani Institute of Management Sciences (BIMS), Rawalpindi, 46300 Pakistan; 5 Center for Biotechnology & Microbiology, University of Swat, Swat, 19130 Pakistan; 6 Diagnostic Department, Nuclear Medicine, Oncology and Radiotherapy Institute (NORI), Islamabad, 44000 Pakistan

**Keywords:** Hepatitis B virus, HBV genotypes, Liver disease progression, PCR

## Abstract

**Background:**

Hepatitis B virus (HBV) genotype and its role in disease progression and patients’ response to antiviral treatment, is not well studied in Pakistan. This comprehensive study was aimed to determine the distribution of HBV genotypes in Pakistan and their possible association with phases of HBV infection.

**Methods:**

A total of 840 HBsAg positive samples was collected and tested for HBV DNA quantity. Samples below 100 IU/ml were excluded from the study. A total of 715 samples representing all the six parts of the country were genotyped by type specific primer PCR method. Clinical data of only 384 patients was compared as the remaining 332 were either receiving antiviral treatment or their infection phase was not confirmed.

**Results:**

Genotype D was found in 509 samples (71.2 %), genotype A in 55 samples (7.7 %) and mixed infection with genotypes A and D in 124 samples (17.3 %). Genotypes B, C and E were identified in less than 1 % of the total samples. Genotype A, D and their mixture (A + D) were compared for severity of HBV infection. Significant differences were not found in distribution of HBV genotypes among different disease stages.

**Conclusion:**

HBV genotype D was the predominant infection in all study areas of Pakistan followed by mixed genotypes infection (A + D) whereas genotype A has 10 times lower prevalence than genotype D. Genotypes B, C, E and F altogether make only 1.5 % of the prevalence. Genotype do not appears to show the severity of liver disease.

## Background

Hepatitis B virus (HBV) infection has been a major health problem as about 400 million people carry hepatitis B surface antigen worldwide [1], out of which, nearly 1 million die every year [2]. The infection rate of HBV has decreased significantly in developed countries like United States of America [3], where the acute HBV infection rate has fallen by 78 % during 1990–2005 [4]. Unfortunately, in developing and underdeveloped countries including Pakistan, the infection rate seems to have not decreased, even to any appreciable level. The failure in tackling the infection has been related to the high cost of antiviral drugs, lack of vaccination and public awareness about infection and the mismanagement to address the problem in Pakistan [5].

Eight HBV genotypes have been established on the basis of 8 % or more nucleotide divergence in the genome. These genotypes are named as A, B, C, D [6], E, F [7, 8], G [9] and H [10] which are common [11], while genotype I [12, 13] and J [14] are also introduced as new genotypes but their status is questionable. HBV genotypes have specific pattern of distribution in different geographic regions and ethnic groups of the world. Genotype A is prevalent in Brazil, USA, Canada, Northwest Europe, South Asia, Central African countries, Tunisia and Benin [15–17]. Genotype B is common in Japan, Taiwan, Philippines, Hong Kong, China, Vietnam, Thailand, Indonesia and United States of America. Genotype C occurs in Australia, Polynesia, Melanesia, Micronesia, Indonesia, China, Hong Kong, Vietnam, Thailand, Japan, Korea, Taiwan, India, Solomon Islands, Brazil and USA. Genotype D is predominant in Mediterranean region, Spain, Albania, Czech Republic, Russia, Turkey, Middle East, Iran, Afghanistan, South Asia, Solomon Islands, Tunisia, Polynesia, Melanesia, Micronesia, Brazil and USA [17–19]. Genotype E is endemic to Africa where it occurs only in some countries of the Western part of the continent while genotype F is widely distributed in new world. It has been reported from Alaska, Argentina, South America, Central America and Polynesia [17, 20]. Genotypes G has been recorded from North America, France and Germany, genotype H from Central America, South America and Mexico while genotypes I and J were reported only from Vietnam and Japan respectively [17].

HBV genotypes have been reported to have significant association with progression of liver diseases, risk of cirrhosis, development of hepatocellular carcinoma (HCC), viral load, HBsAg sero-clearance, HBeAg sero-conversion and response to antiviral therapy [21]. Co-infection with two different virus genotypes has been reported to be associated with worse prognosis of the disease. Some studies showed that co-infection with HBV/B and HBV/C is associated with high viral load and more severe liver disease as compared to single genotype infection [22, 23]. The patients with HBV/A have been reported to be more sensitive to interferon α treatment as compared to those infected with HBV/D [24] while the patients having infection with HBV/B have a higher response rate to interferon α treatment as compared to the patients infected with HBV/C [25]. Moreover, infection with genotype C alone was also found to be associated with significantly higher risks of liver cirrhosis and hepatocellular carcinoma as compared to genotype B infection [26, 27] while HBV/B shown an earlier HBsAg sero-clearance rate as compared to HBV/C [28]. In another study, HBV/A was found to have significantly higher sero-conversion rate of HBeAg as compared to HBV/B, HBV/C, HBV/D and HBV/F [29]. From all the studies mentioned above it’s quite clear that genotypes influence disease condition as well as response to treatment.

The picture regarding distribution of HBV genotypes in Pakistan and its association with disease stage has not been clearly understood yet. In fact, all of the available studies are mostly confined to specific cities/areas like Karachi (Sind) and Lahore (Punjab) and none of them contain data from other parts of the country. A vast range of areas from different regions of Pakistan still remains unexplored for HBV genotypes. Moreover, the existing state of knowledge regarding the distribution and prevalence of HBV genotypes in Pakistan seems to be sketchy and questionable too. For example, four studies [5, 30–32] are in agreement that genotype D infection is most prevalent in the country while three studies [18, 33, 34] reported that genotype C is the most common ones in Pakistan. Yet in an another study, genotype C and D have been cited as most common HBV genotypes in Pakistan [35]. In view of the presence of the inadequate data, it was felt that a comprehensive study is required to determine the current distribution of HBV genotypes in all the six provinces of Pakistan and their possible association with different phases of HBV infection.

## Methods

### Patients

**Fig. 1:**
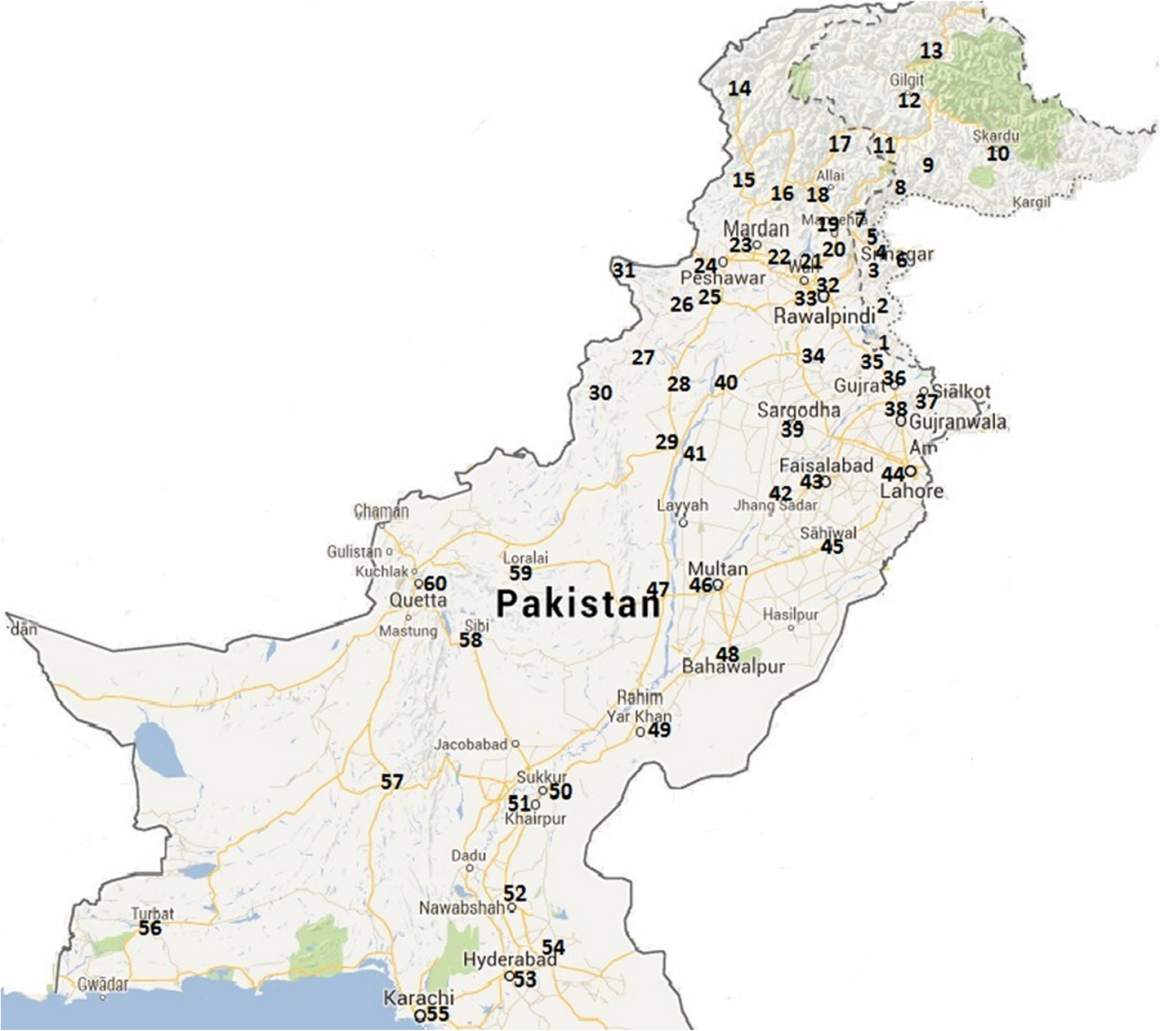
The map shows the sampling sites in all regions of Pakistan. Sites are indicated with numerals. Sites: 1. Mirpur; 2. Kotli; 3. Palandari; 4. Rawalakot; 5. Bagh; 6. Kahuta; 7. Muzaffarabad; 8. Neelum; 9. Astor; 10. Sikardu; 11. Diamir; 12. Gilgit; 13. Hunza; 14. Chitral; 15. Dir; 16. Swat; 17. Kohistan; 18. Batgram; 19. Mansehra; 20. Abottabad; 21. Haripur; 22. Nowshera; 23. Mardan; 24. Peshawar; 25. Kohat; 26. Hangu; 27. Bannu; 28. LakkiMarwat; 29. D I Khan; 30. Wana; 31. Parachinar; 32. Islamabad; 33. Rawalpindi; 34; Chakwal; 35. Jhelum; 36. Gujrat; 37. Sialkot; 38. Gujranwala; 39. Sargodha; 40. Mianwali; 41. Bhakkar; 42. Jhang; 43. Faisalabad; 44. Lahore; 45. Sahiwal; 46. Multan; 47. D.G. Khan; 48. Bahawalpur; 49. Rahim Yar Khan; 50. Sukhar; 51. Khairpur; 52. Nawabshah; 53. Hyderabad; 54. Mirpur Khas; 55. Karachi; 56. Turbat; 57; Khuzdar; 58. Sibi; 59. Loralai; 60. Quetta. Regions: 1–8, Azad Jammu & Kashmir; 9–13, Gilgit-Baltistan; 14–31, Khyber Pakhtunkhwa; 32, Federal Capital Territory of Islamabad (FCT); 33–49, Punjab; 50–55, Sind; 56–60, Baluchitan

Eight hundred and forty (840) samples from HBsAg positive patients were collected during October 2010 to March 2013 from different locations of six regions of Pakistan which include Punjab and Federal Capital Territory of Islamabad (*n* = 359); Khyber Pakhtoonkhwa along with Federally Administered Tribal Areas (*n* = 115); Azad Jammu and Kashmir (*n* = 81); Gilgit-Baltistan (*n* = 111); Sind province including Karachi city (*n* = 107); and Baluchistan (*n* = 67). The sampling sites and the details of patients are given in Fig. 1 and Table 1 respectively. All the patients were diagnosed as chronic HBV carriers. Serum samples were acquired from the different hospitals and diagnostic laboratories of the above regions where they were originally collected for routine diagnostic purposes. All the available data of clinical and demographic importance were collected at the time of sample collection by filling out a questionnaire. All the samples were then brought to Nuclear Medicine, Oncology and Radiotherapy Institute (NORI), Islamabad and stored at −25 °C for further analysis. The samples were tested for positivity and quantity of HBV DNA. Only the samples having more than 100 IU/ml of viral load were subjected to HBV genotyping (Table 1). The samples with lower than 100 IU/ml of viral load were excluded because their genotyping was not successful in most of the cases. All the routine tests including HBsAg, ALT, and anti-HBe were performed in Central Laboratories, NORI using commercially available kits (Abott Diagnostics, USA).

**Table 1:** Samples collected and selected for HBV genotyping from different regions of the country

Total no of samples (No of male and female samples)							
	Punjab and FCT	KP and FATA	AJK	Gilgit-Baltistan	Sind	Baluchistan	Total
Total collected	359 (243 + 116)	115 (78 + 37)	81 (50 + 31)	111 (83 + 28)	107 (68 + 39)	67 (39 + 28)	840 (561 + 279)
Negative HBV DNA	29 (18 + 11)	7 (5 + 2)	6 (4 + 2)	8 (6 + 2)	13 (7 + 6)	6 (4 + 2)	69 (44 + 25)
<100 IU/ml	19 (11 + 8)	9 (6 + 3)	7 (4 + 3)	10 (6 + 4)	10 (7 + 3)	1 (0 + 1)	56 (34 + 22)
Total Genotyped	311 (214 + 97)	99 (67 + 32)	68 (42 + 26)	93 (71 + 22)	84 (54 + 30)	60 (35 + 25)	715 (483 + 232)

*Abbreviations*: *FCT* federal capital territory, *KP* Khyber Pkhtoonkhwa, *FATA* federally administered tribal areas, *AJK* Azad Jammu and Kashmir

The stage of HBV infection was primarily assigned by the local physician and further confirmed by the status of different HBV markers and biochemical tests. Only 384 patients were included in the study of genotypes influence on disease stage. All the patients (331) who had already received antiviral treatment or had co-infection with HIV, HCV or HDV were excluded from this part of study. The liver disease stage was primary assigned by classified gastroenterologists and then the disease phase was further confirmed on the base of patients’ HBV viral load, ALT levels, HBeAg status and the presence or absence of anti HBe antibodies (Table 3). The study was approved by institutional ethics committee of PMAS Arid Agriculture University Rawalpindi and each patient gave written consent.

### HBV DNA extraction and quantification

HBV DNA was extracted from the plasma according to manufacturer’s protocol using “Instant Virus DNA kit” (AJ Roboscreen, Analytikajena Biosolutions, GmbH, Germany) while the DNA was quantified using RoboGene® HBV Quantification kit (AJ Roboscreen, analytikajena Biosolutions, GmbH, Germany) according to manufacturer’s instructions.

### HBV genotyping

**Fig. 2:**
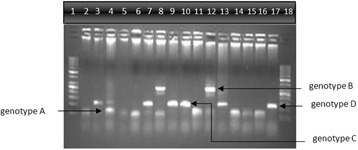
Amplified PCR products on 2 % gel showing bands for four genotypes. Lanes 2, 4, 6, 8, 10, 12, 14 and 16: the product bands of mix 1. Lanes 3, 5, 7, 9, 11, 13, 15 and 17: the product bands of mix 2. Lanes 1 and 18: 50 bp DNA ladder. lanes 2 + 3: genotype D. lanes 4 + 5: genotype A. lanes 6 + 7: genotype A + D. lanes 8 + 9: genotype A + B + D. lanes 10 + 11: genotype C and a nonspecific band. lanes 12 + 13: genotype B + D. lanes 14 + 15: genotype A. lanes 16 + 17; Genotype A + D

The genotyping was performed using PCR method with genotype specific primers described by Naito et al. [36] with some modifications in cycling profile and PCR constituents. For regular PCR, 10 μl of GoTaq® Green Master mix (Promega, USA), 1 μl each of universal primers P1A and S1-2, 2 μl of ddH_2_O and 6 μl of diluted template (DNA) were used to make 20 μl of reaction volume. The thermal profile was; 10 min at 95 °C, then 30 cycles of; 94 °C for 20 s, 55 °C for 20 s and 72 °C for 1 min, then another 7 min at 72 °C (ABI 9700, USA). Two mixes were used in nested PCR. Mix 1 had the forward primers for genotype A, B and C with a common reverse primer while mix two had reverse primers for genotypes D, E and F with common forward primer. Two μl of product from 1st round of PCR were added to each mix as DNA template with 8 μl of GoTaq® Green Master mix, 8 μl of ddH2O and 1 μl of each primer to make 22 μl of final volume. The cycling profile for nested PCR was; 95 °C for 10 min, then 40 cycles of 94 °C for 45 s, 63 °C for 20 s, and 72 °C for 60 s, and then another 7 min at 72 °C (ABI 9700, USA). Products were run on 2 % agarose gel which was stained in a solution of ethidium bromide, and then observed under ultraviolet fluorescence (BioRad Gel Doc-XR, USA). Amplified products in mix 1 were 68 bp, 281 bp and 122 bp in size for genotypes A, B and C respectively while the product sizes in mix 2 were 119 bp, 167 bp and 97 bp for genotypes D, E and F respectively. The product’s size was estimated with reference to a 50-bp DNA ladder (GeneRuler™ Fermentas) and the genotype was determined for each sample according to the sizes of DNA product obtained in both mixes (Fig. 2). The term untypable was assigned to those samples which had higher than 100 IU/ml of viral load but were not successfully genotype or their genotype was not identified by the method.

### Statistical analysis

Chi square test was applied to evaluate the relationship HBV genotype and HBV infection phase with each other as well as with patients’ gender while one way analysis of variance (ANOVA) was performed to analyze the relationship of HBV genotype and HBV infection phase with the age of patients. A value of *p* less than 0.05 was considered as statistically significant. SPSS version-16.0 was used for all the analyses.

## Results

Out of total 840 samples, 125 were excluded from the study as HBV DNA was not detected in them. The remaining 715 samples were further analyzed (Table 1).

### Regional distribution of HBV genotypes

Out of the 715 samples taken for genotyping, 699 were successfully genotyped while 16 (2.2 %) remained untypable. HBV genotypes A, B, C, D and E were detected in 55 (7.7 %), 4 (0.6 %), 6 (0.8 %), 509 (71.2 %) and 1 (0.1 %) samples respectively (Table 2). A total of 124 (17.3 %) patients were infected with more than one genotype i.e. mix genotype infection. The mix genotype infection comprised six combinations in different proportions which were - A + B (0.3 %), A + D (13.7 %), B + D (1.9 %), C + D (1.0 %), E + D (0.1 %) and A + B + D (0.3 %).

**Table 2:** Distribution of HBV genotypes and their combinations in the samples taken from different regions of Pakistan. The numbers and proportions (%) are given against each genotype or the genotypic combination

Genotype	KP and FATA	AJK	Gilgit-Baltistan	Sind	Punjab and FCT	Baluchistan	Total
A	7 (7)	3 (4.4)	3 (3.2)	9 (10.7)	31 (10)	2 (3.3)	55 (7.7)
B	2 (2)	0	1 (1.1)	0	1 (0.3)	0	4 (0.6)
C	3 (3)	1 (1.5)	0	0	1 (0.3)	1 (1.7)	6 (0.8)
D	67 (68)	56 (82.3)	75 (80.6)	61 (72.6)	197 (63.3)	53 (88.3)	509 (71.2)
E	0	0	0	1 (1.2)	0	0	1 (0.1)
Mix	18 (18)	8 (11.8)	11 (12.8)	11 (13)	73 (23.5)	3 (5)	124 (17.3)
A + B	2 (2.0)	0	0	0	0	0	2 (0.3)
A + D	9 (9.1)	5 (7.3)	4 (4.3)	9 (10.7)	68 (21.8)	3 (5.0)	98 (13.7)
B + D	2 (2.0)	0	6 (6.4)	2 (2.3)	4 (1.3)	0	14 (1.9)
C + D	4 (2.0)	3 (4.4)	0	0	0	0	7 (1.0)
E + D	0	0	0	0	1 (0.3)	0	1 (0.1)
A + B + D	1 (1.0)	0	1 (1.1)	0	0	0	2 (0.3)
Untypable	2 (2.0)	0	3 (3.2)	2 (2.3)	8 (2.5)	1 (1.7)	16 (2.2)
Total	99	68	93	84	311	60	715

**Fig. 3:**
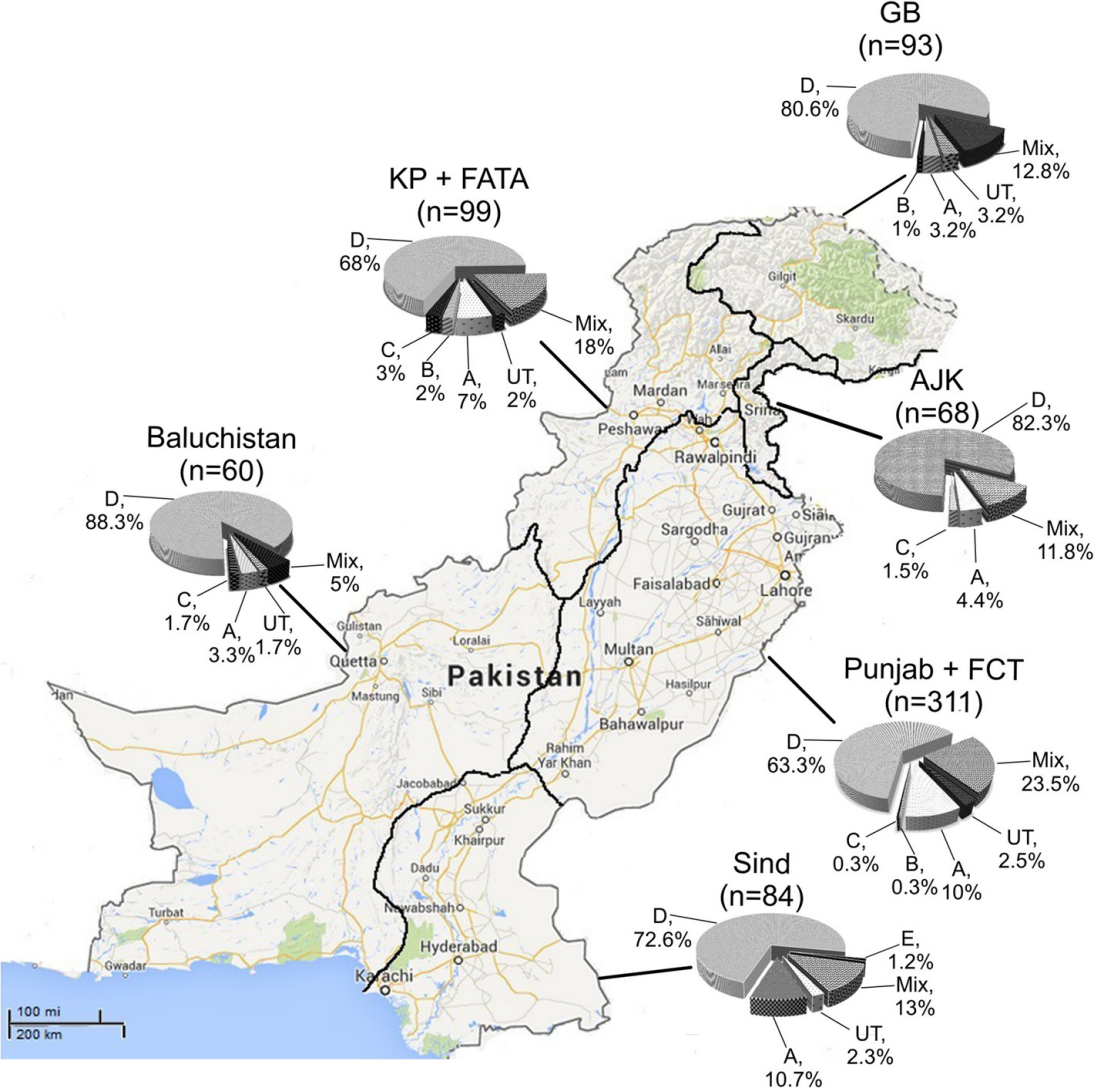
Genotype prevalence of hepatitis B virus in all regions of the country. UT stands for un-typable and mix means all the infections with more than one genotypes

Concerning the regions included in this study, genotype D was always the predominant one with the prevalence ranging from 63.3 to 88.3 % (Table 2). Genotype A and the mix genotype combination A + D was also present in all regions. The prevalence of genotype A, D and mix genotypes respectively was 10 %, 63.3 % and 23.5 % in Punjab and FCT region; 7 %, 68 % and 18 % in KP and FATA region; 4.4 %, 82.3 % and 11.8 % in AJK region; 3.2 %, 80.6 % and 12.8 % in Gilgilt-Baltistan region; 10.7 %, 72.6 % and 13 % in Sind region; 3.3 %, 88.3 % and 5 % in Baluchistan region (Fig. 3).

Multiple genotype infections presented the second more prevalent group of genotypes with 17.3 % of the total samples infected with 6 different combinations of genotypes (Table 2). Mix infection with A + D genotype was the major combination found among multiple genotype infections which was 98/124 of the samples. Other mix genotype combinations identified were B + D, C + D, A + B, A + B + D and E + D which were 1.9 %, 1.0 %, 0.3 %, 0.3 % and 0.1 % of the samples respectively. None of the combinations except A + D was found in more than 2 % of the samples. This combination was detected from all parts of the Pakistan, though it was highest in Punjab and FCT region (21.8 %) as compared to all other parts (Table 2).

### Distribution HBV genotypes in different phases of disease

All statistical comparisons performed for genotypes A, D and A + D as the other genotypes and their combinations in mix infections were rare (Table 2). Out of total 715 patients included in the study, 331 (46.3 %) had either already received antiviral treatment or had co-infection with HIV, HCV or HDV or had genotype other than A and D. Thus the remaining 384 (53.7 %) patients who were not exposed to any treatment till the time of sample collection were classified in four different phases of liver disease: 56 (14.6 %) were in immune control phase, 89 (23.2 %) in immune tolerant phase, 153 (39.8 %) in immune clearance phase and 86 (22.4 %) in immune escape phase (Table 3). None of the patients had liver cirrhosis or hepatocellular carcinoma in this study. The mean age of the patients and gender ratio was statistically equal among all the disease phases. Genotype D (*n* = 279) was the most prevalent in all groups of patients with 36 patients in immune control group, 67 in immune tolerant group, 112 in immune clearance and 64 in immune escape group. The second more prevalent was the mix infection A + D (*n* = 62) with 13, 12, 26 and 11 cases in immune control, immune tolerant, immune clearance and immune escape groups respectively. Genotype A (*n* = 43) was the least prevalent with 7, 10, 15 and 11 cases in immune control, immune tolerant, immune clearance and immune escape phases respectively. The differences in distribution of genotypes in disease phases were not statistically significant. Similarly, there were no statistical differences of age and gender among the three genotype groups compared (Table 3).

**Table 3:** Characteristics of patients infected with different HBV genotypes (*n* = 384)

Characteristics		Genotype A	Genotype D	Genotype A + D	Sig.
Number of patients (%)		43 (11.2)	279 (72.7)	62 (16.1)	
Age (Mean ± SD)		32.4 ± 11.7	34.1 ± 12.1	35.1 ± 12.8	NS^a^
Gender (M/F)		32/11	202/77	40/22	NS^b^
Immune Control (*N* = 56)	normal ALT, HBeAg negative, anti HBe positive, low viral load	7 (16.3 %)	36 (12.9 %)	13 (21.0 %)	NS^b^
Immune Tolerant (*N* = 89)	normal ALT, HBeAg positive, anti HBe negative, high viral load	10 (23.2 %)	67 (24.0 %)	12 (19.3 %)	NS^b^
Immune Clearance (*N* = 153)	elevated ALT, HBeAg positive, high viral load	15 (34.9 %)	112 (40.1 %)	26 (41.9 %)	NS^b^
Immune Escape (*N* = 86)	Normal or elevated ALT, HBeAg negative, anti HBe positive, High HBV DNA,	11 (25.6 %)	64 (22.9 %)	11 (17.7 %)	NS^b^

^a^One way Analysis of Variance, ^b^Pearson Chi Square Test, NS for non significant

## Discussion

The present study is the first of its kind which included a cohort of 715 patients with chronic HBV infection from all six regions of Pakistan. The study analyzed the patients for HBV genotypes and their possible associations with liver disease severity in 384 consecutive chronic HBV carriers. According to the results, genotypes A, B, C, D, E and mix genotype infections with 6 different genotypic combinations are present in Pakistan, but the majority of patients (92.6 %) were infected with either genotype D, or genotype A or a mixture of both (A + D). The pre-dominant genotype in all regions was D followed by genotype A and mix genotype infection with A + D. Genotypes B, C, E and F were rare and collectively form only 1.5 % of the total prevalence.

HBV genotypes were not studied exclusively from all over the Pakistan so far. Table 4 summarizes some available data on the topic from Pakistan along with the results of present study. Most of the available data are from Karachi and Lahore or Punjab and Sind while none of the study has explored the regions of FATA, Gilgit-Baltistan, AJK, some parts of Khyber Pakhtoonkha province and most of the Baluchistan province. Though three studies carried out by Awan et al. [18], Alam et al. [30] and Idrees et al. [34] investigated the provinces of KP and Baluchistan additionally but the number of samples taken were not satisfactory to conclude the genotype distribution in these provinces (Table 4). None of the studies included the regions of Gilgit-Baltistan, AJK and FATA. Two of these three [18, 34] studies reported genotype C as predominant while one [30] found genotype D as predominant one. Genotype B which was not reported from Pakistan in any other study has also been reported in all three of these studies from 18 to 25 % of the samples. These results are contradictory to these studies.

**Table 4:** The prevalence of different HBV genotypes in our study compared to previously published studies

Source	Place of sampling	Sample size	Proportion of genotype as percent of total samples								
			A	B	C	D	E	F	Mix	Mixed combinations	UT
Hanif et al., 2013 [5]	Karachi, Islamabad/Rawalpindi	200 (40, 160)	10	-	-	59	-	-	31	A + D	-
Awan et al., 2010 [18]	Punjab, KP, Sind, Baluchistan	300 (222, 36, 26, 15)	14	18	28	13	0.6	1.3	16	A + B + D, A + D + F, A + C, A + D, A + E, A + F, B + C, B + E, C + D	10.3
Ahmed et al., 2009 [39]	Punjab, Sind	236	0.8	-	5.9	93.2	-	-	-	-	-
Baig et al., 2009 [32]	Karachi	315	20	-	-	70	-	-	10	A + D	-
Noorali et al., 2008 [31]	Karachi	180	-	-	-	84	-	-	16	B + D	-
Hakim et al., 2008 [38]	Karachi	180	-	-	-	84	-	-	16	B + D	-
Alam et al., 2007 [30]	Punjab, KP, Sind Baluchistan	110 (30, 28, 25, 18)	4.5	24.5	-	60	-	-	2.7	A + D, B + D	8.2
Abbas et al., 2006 [37]	Karachi	109	-	-	-	98.2	-	-	1.8	A + D	-
Idrees et al., 2004 [34]	Punjab, KP, Sind Baluchistan	112 (Details not available)	21.4	17.9	41.1	8.0	-	-	7.1	Details not available	4.5
Naaz, 2001 [33]	Lahore	12	-	-	75	25	-	-	-	-	-
This study	Punjab, KP, Sind, Baluchistan, AJK, Gilgit-Baltistan	715 (311, 99, 84, 60, 68, 93)	7.7	0.6	0.8	71.2	0.1	-	17.3	A + D, B + D, C + D, A + B, A + B + D, E + D	2.2

*UT* stands for Un-typable

All the studies confined to the samples from Punjab and Sind reported genotype D as predominant except one [33] which reported prevalence of genotype C and D as 75 and 25 % respectively. The remaining six studies [5, 31, 32, 37–39] reported genotype D as the commonest one in Pakistan with percentage distribution of 59, 84, 70, 98.2, 84 and 93.2 % respectively (Table 4). The results of present study representing all parts of the country with significant number of samples is in agreement with the later group [5, 31, 32, 37–39] that reported genotype D as the most prevalent HBV genotype in Pakistan. Genotype A was found to be the second more prevalent single genotype infection with 7.7 %. Genotype A was also reported in most of the previous studies [5, 18, 30, 32, 34, 39] in different proportions ranging from 0.8 to 21.4 % and a mean prevalence of 11.7 % (Table 4). Genotype B was present only in 0.6 % of present study samples while it was reported in proportions ranging from 18 to 24.5 % by previous studies from Pakistan [18, 30, 34]. None of the other studies reported genotype B infection, however mix infections involving genotype B were reported by some studies [18, 30, 31, 38] as high as 16 % of the samples (Table 4). Genotype C was detected in only 0.8 % of the samples in our study while three previous studies [18, 33, 34] have reported it as predominant genotype in Pakistan, whereas one study [39] reported it as second more prevalent genotype in the country. Genotype C was not reported by any of the other six studies (Table 4). Genotype E and F were not reported by any previous study except [18] which reported them in the proportions of 0.6 and 1.3 %. As already mentioned, genotypes E and F are endemic to Africa and the New World respectively [17, 20] and the fact that they were found in only 1 case (genotype E) was quite expected. Present study thus supports the conclusion that not only genotypes E and F but also genotypes B and C were rare in Pakistan (Table 4).

The relationship of HBV genotypes with liver disease is still not very clear. Although some studies have reported that HBV genotype influence the severity of liver disease and course of chronic HBV infection [26, 28, 40–44]. Some other studies showed that HBV genotype had no influence on course of HBV infection [43, 45, 46]. Present study is in concordance with the later group [43, 45, 46]. Most of the studies compared genotype B and C in Asia and reported that the latter is more associated to severe liver disease than the former. On the other hand the genotype B is associated with an early HBeAg sero-conversion, more sustained remission after HBeAg sero-conversion, less active hepatic necroinflammation, a slower rate of progression to cirrhosis, and a lower rate of hepatocellular carcinoma (HCC) development as compared to genotype C [26, 28, 41, 43, 44]. A study from Pakistan compared genotype A, D and A + D for their association with complex liver diseases and concluded that genotype A is associated with more complex liver disease [47]. However, present study did not find any association of genotypes with disease stage. The relatively high prevalence of mix infection with genotypes A + D is a confirmation of the data reported by several previous findings about HBV genotyping in Pakistan [5, 18, 30, 32, 34, 37].

## Conclusions

In conclusion, it has been observed in the present study that the distribution of HBV genotypes was almost similar in all the regions of Pakistan. Genotype D was the most common one (71.2 %) followed by mix infection with genotypes A and D (17.3 %) and genotype A (7.7 %). Although genotypes B, C, E and F were also detected but their prevalence was very low (1.5 %). Furthermore, genotype was not found to have any significant influence on liver disease progression in Pakistan.

## Abbreviations

AJK: Azad Jammu and Kashmir
ANOVA: Analysis of Variance
bp: Base Pairs
ddH_2_O: Double Distilled Water
DNA: Deoxy ribo nucleic acid
FATA: Federally Administered Tribal Areas
FCT: Federal Capital Territory
HBsAg: Hepatitis B Surface Antigen
HBV: Hepatitis B Virus
HCC: Hepatocellular Carcinoma
HEC: Higher Education Commission
IU/ml: International Unit Per Milli Liter
KP: Khyber Pakhtoonkha
NORI: Nuclear Medicine, Oncology and Radiotherapy Institute
PCR: Polymerase Chain Reaction
PMAS: Pir Mehr Ali Shah
SPSS: Statistical Package for Social Sciences
USA: United States of America
°C: Centigrade
